# Supplementary material for: The novel lncRNA lnc-NR2F1 is pro-neurogenic and mutated in human neurodevelopmental disorders
Source: eLife. 2019 Jan 10;8:e41770. doi: 10.7554/eLife.41770 (PMC6380841; doi:10.7554/eLife.41770)
Supplement: Supplementary file 1. — Related to Figure 2 (A) Summary of diagnosis for previously reported patients, including patient CMS12200 described in this study. Highlighted in grey are the shared diagnostic features across patients. Adapted figure (Al-Kateb et al., 2013). [file elife-41770-supp1.docx]

|  | CMS12200 | Al-Kateb et al, 2013 | Brown et al, 2009 | Cardoso et al, 2009 patient 1 | Cardoso et al, 2009 patient 2 | Cardoso et al, 2009 patient 3 |
| --- | --- | --- | --- | --- | --- | --- |
| Age (yrs) | 2.7 | 8 | 4 | 7 | 5 | 5 |
| Gender | Male | Male | Female | Male | Female | Male |
| Genomic locus affected | 5q15  [92808914]  12q15  [61902666] | 5q15  [92,742,875-93,324,350] | 5q15  [92,612,533-92,972,632]  5q33  [153,390,771-153,844,258] | 5q15  [87,086,298-95,538,640] | 5q15  [87,086,298-357; 95,538,640-699] | 5q15  [88,641,401-94,876,462] |
| Type of chromosomal abnormality | Balanced chromosomal translocation  (5;12)(q15;q15)  9 bp deletion at 5q15  12 bp deletion at 12q15 | deletion  582 Kb | Balanced paracentric  chromosome 5 inversion inv(5)(q15q33.2)  ~400-500 kb | der(5)  del(5)(q14;21)t(1,5)(q31;q14)  17Mb | del(5)(q14.3-q15)  8.4Mb | del(5)(q14.2q15)  6.3Mb |
| Inheritance | paternally inherited | de novo | de novo | de novo | de novo | de novo |
| Genes affected | NR2F1-AS | NR2F1-AS, NR2F1, FAM172A, POU5F2 | NR2F1-AS and NR2F1 | >10 genes | >10 genes | >10 genes |
| Developmental delay | + | + | + | + | + | + |
| Congenital malformations  Hypertelorism | N/A | + | + | + | - | + |
| Periventricular heterotopia | N/A | - | - | + | + | + |
| Ophthalmological condition | + | + | + | + | - | - |
| Hypertelorism | N/A | - | - | + | + | - |
| High arched eyebrows | N/A | - | - | + | + | - |
| Abnormal slanting of  palpebral fissures | N/A | - | + | + | - | - |
| Micrognathia | N/A | - | + | - | + | - |
| Abnormal ears | N/A | + | + | - | - | - |
| Nasal abnormalities | N/A | + | + | + | + | - |
| Philtrum | N/A | + | - | + | + | - |
| Thin lips | N/A | + | - | - | + | - |
| High forehead | N/A | - | - | + | + | - |
| Head size | 5th-10th centile | 80th centile | N/A | N/A | N/A | >98th centile |
| Fifth finger clinodactyly bilaterally | + | N/A | N/A | N/A | N/A | N/A |
| Seizures | - | - | - | + | + | + |
| Hearing loss | N/A | + | + | N/A | N/A | N/A |
| Language delay / no language skills | + | N/A | N/A | + | + | + |
| Fine motor skills abnormalities | N/A | + | + | N/A | N/A | N/A |
| Feeding difficulties | N/A | + | + | N/A | N/A | N/A |
