# Supplementary material for: The novel lncRNA lnc-NR2F1 is pro-neurogenic and mutated in human neurodevelopmental disorders
Source: eLife. 2019 Jan 10;8:e41770. doi: 10.7554/eLife.41770 (PMC6380841; doi:10.7554/eLife.41770)
Supplement: Supplementary file 6. [file elife-41770-supp6.docx]

**Sequences used for discover microhomology (Figure 2A)**

Exon a

Human NR2F1-201 (mapped to ENSMUST00000123998 first exon)

ACAA[G](https://uswest.ensembl.org/Homo_sapiens/ZMenu/TextSequence?db=core;factorytype=Location;g=ENSG00000237187;r=5:93399495-93595784;t=ENST00000503134;v=rs951421933;vf=218952974)T[C](https://uswest.ensembl.org/Homo_sapiens/ZMenu/TextSequence?db=core;factorytype=Location;g=ENSG00000237187;r=5:93399495-93595784;t=ENST00000503134;v=rs191159087;vf=45682411)GGGAGTC[G](https://uswest.ensembl.org/Homo_sapiens/ZMenu/TextSequence?db=core;factorytype=Location;g=ENSG00000237187;r=5:93399495-93595784;t=ENST00000503134;v=rs1012257099;vf=279806843)G[C](https://uswest.ensembl.org/Homo_sapiens/ZMenu/TextSequence?db=core;factorytype=Location;g=ENSG00000237187;r=5:93399495-93595784;t=ENST00000503134;v=rs568585919;vf=101050361)GACCGCTTA[G](https://uswest.ensembl.org/Homo_sapiens/ZMenu/TextSequence?db=core;factorytype=Location;g=ENSG00000237187;r=5:93399495-93595784;t=ENST00000503134;v=rs901732725;vf=169248456)TGACGAG[C](https://uswest.ensembl.org/Homo_sapiens/ZMenu/TextSequence?db=core;factorytype=Location;g=ENSG00000237187;r=5:93399495-93595784;t=ENST00000503134;v=rs544529070;vf=77080958)GATGATTTTAAAGAG[C](https://uswest.ensembl.org/Homo_sapiens/ZMenu/TextSequence?db=core;factorytype=Location;g=ENSG00000237187;r=5:93399495-93595784;t=ENST00000503134;v=rs1039782617;vf=307340875)[A](https://uswest.ensembl.org/Homo_sapiens/ZMenu/TextSequence?db=core;factorytype=Location;g=ENSG00000237187;r=5:93399495-93595784;t=ENST00000503134;v=rs548662642;vf=81199520)GAATAAAA

GGTTTTCCCGT[G](https://uswest.ensembl.org/Homo_sapiens/ZMenu/TextSequence?db=core;factorytype=Location;g=ENSG00000237187;r=5:93399495-93595784;t=ENST00000503134;v=rs1007893398;vf=275441859)CCTGCCCCAC[A](https://uswest.ensembl.org/Homo_sapiens/ZMenu/TextSequence?db=core;factorytype=Location;g=ENSG00000237187;r=5:93399495-93595784;t=ENST00000503134;v=rs1007379828;vf=274928127)G[C](https://uswest.ensembl.org/Homo_sapiens/ZMenu/TextSequence?db=core;factorytype=Location;g=ENSG00000237187;r=5:93399495-93595784;t=ENST00000503134;v=rs890907595;vf=158420072)[C](https://uswest.ensembl.org/Homo_sapiens/ZMenu/TextSequence?db=core;factorytype=Location;g=ENSG00000237187;r=5:93399495-93595784;t=ENST00000503134;v=rs888902886;vf=156414726)CCTCCAG[C](https://uswest.ensembl.org/Homo_sapiens/ZMenu/TextSequence?db=core;factorytype=Location;g=ENSG00000237187;r=5:93399495-93595784;t=ENST00000503134;v=rs1054203229;vf=321766012)TCGAATAATGGAGAACTGTCAATGCA

CTCCGCGAGCGG[T](https://uswest.ensembl.org/Homo_sapiens/ZMenu/TextSequence?db=core;factorytype=Location;g=ENSG00000237187;r=5:93399495-93595784;t=ENST00000503134;v=rs867826612;vf=151315248)GGCCCATTG[T](https://uswest.ensembl.org/Homo_sapiens/ZMenu/TextSequence?db=core;factorytype=Location;g=ENSG00000237187;r=5:93399495-93595784;t=ENST00000503134;v=rs929833400;vf=197357760)A[C](https://uswest.ensembl.org/Homo_sapiens/ZMenu/TextSequence?db=core;factorytype=Location;g=ENSG00000237187;r=5:93399495-93595784;t=ENST00000503134;v=rs902849413;vf=170365493)[G](https://uswest.ensembl.org/Homo_sapiens/ZMenu/TextSequence?db=core;factorytype=Location;g=ENSG00000237187;r=5:93399495-93595784;t=ENST00000503134;v=rs1041340197;vf=308898954)CAGCTGATGGC[G](https://uswest.ensembl.org/Homo_sapiens/ZMenu/TextSequence?db=core;factorytype=Location;g=ENSG00000237187;r=5:93399495-93595784;t=ENST00000503134;v=rs529081661;vf=61689564)A[G](https://uswest.ensembl.org/Homo_sapiens/ZMenu/TextSequence?db=core;factorytype=Location;g=ENSG00000237187;r=5:93399495-93595784;t=ENST00000503134;v=rs575773220;vf=108211359)GAGGCG[A](https://uswest.ensembl.org/Homo_sapiens/ZMenu/TextSequence?db=core;factorytype=Location;g=ENSG00000237187;r=5:93399495-93595784;t=ENST00000503134;v=rs917482904;vf=185003482)C[T](https://uswest.ensembl.org/Homo_sapiens/ZMenu/TextSequence?db=core;factorytype=Location;g=ENSG00000237187;r=5:93399495-93595784;t=ENST00000503134;v=rs1038177337;vf=305735120)GGGGGTAGCAG

GGGCTCCTTGCC[C](https://uswest.ensembl.org/Homo_sapiens/ZMenu/TextSequence?db=core;factorytype=Location;g=ENSG00000237187;r=5:93399495-93595784;t=ENST00000503134;v=rs566655948;vf=99127527)T[C](https://uswest.ensembl.org/Homo_sapiens/ZMenu/TextSequence?db=core;factorytype=Location;g=ENSG00000237187;r=5:93399495-93595784;t=ENST00000503134;v=rs546618037;vf=79162337)[G](https://uswest.ensembl.org/Homo_sapiens/ZMenu/TextSequence?db=core;factorytype=Location;g=ENSG00000237187;r=5:93399495-93595784;t=ENST00000503134;v=rs991938012;vf=259481524)TCCCTATGCCCAAATAAAC[G](https://uswest.ensembl.org/Homo_sapiens/ZMenu/TextSequence?db=core;factorytype=Location;g=ENSG00000237187;r=5:93399495-93595784;t=ENST00000503134;v=rs533237891;vf=65830611)CG[C](https://uswest.ensembl.org/Homo_sapiens/ZMenu/TextSequence?db=core;factorytype=Location;g=ENSG00000237187;r=5:93399495-93595784;t=ENST00000503134;v=rs937519374;vf=205046040)AAAC[G](https://uswest.ensembl.org/Homo_sapiens/ZMenu/TextSequence?db=core;factorytype=Location;g=ENSG00000237187;r=5:93399495-93595784;t=ENST00000503134;v=rs910008838;vf=177527164)C[G](https://uswest.ensembl.org/Homo_sapiens/ZMenu/TextSequence?db=core;factorytype=Location;g=ENSG00000237187;r=5:93399495-93595784;t=ENST00000503134;v=rs984225212;vf=251766371)CT[C](https://uswest.ensembl.org/Homo_sapiens/ZMenu/TextSequence?db=core;factorytype=Location;g=ENSG00000237187;r=5:93399495-93595784;t=ENST00000503134;v=rs563764936;vf=96247013)CCGT[C](https://uswest.ensembl.org/Homo_sapiens/ZMenu/TextSequence?db=core;factorytype=Location;g=ENSG00000237187;r=5:93399495-93595784;t=ENST00000503134;v=rs550180498;vf=82711783)TT[C](https://uswest.ensembl.org/Homo_sapiens/ZMenu/TextSequence?db=core;factorytype=Location;g=ENSG00000237187;r=5:93399495-93595784;t=ENST00000503134;v=rs530387534;vf=62990656)TGG

TC[A](https://uswest.ensembl.org/Homo_sapiens/ZMenu/TextSequence?db=core;factorytype=Location;g=ENSG00000237187;r=5:93399495-93595784;t=ENST00000503134;v=rs951523440;vf=219054526)GTTAAAAG[G](https://uswest.ensembl.org/Homo_sapiens/ZMenu/TextSequence?db=core;factorytype=Location;g=ENSG00000237187;r=5:93399495-93595784;t=ENST00000503134;v=rs1025664639;vf=293218502)TGAAACGAACACGATTAAAATAGACCAAAAG[A](https://uswest.ensembl.org/Homo_sapiens/ZMenu/TextSequence?db=core;factorytype=Location;g=ENSG00000237187;r=5:93399495-93595784;t=ENST00000503134;v=rs928728271;vf=196252298)AAA[G](https://uswest.ensembl.org/Homo_sapiens/ZMenu/TextSequence?db=core;factorytype=Location;g=ENSG00000237187;r=5:93399495-93595784;t=ENST00000503134;v=rs977521973;vf=245061126)AAAGAAA[A](https://uswest.ensembl.org/Homo_sapiens/ZMenu/TextSequence?db=core;factorytype=Location;g=ENSG00000237187;r=5:93399495-93595784;t=ENST00000503134;v=rs914648291;vf=182168040)[G](https://uswest.ensembl.org/Homo_sapiens/ZMenu/TextSequence?db=core;factorytype=Location;g=ENSG00000237187;r=5:93399495-93595784;t=ENST00000503134;v=rs914648291;v=rs967428126;vf=182168040;vf=234964119)[A](https://uswest.ensembl.org/Homo_sapiens/ZMenu/TextSequence?db=core;factorytype=Location;g=ENSG00000237187;r=5:93399495-93595784;t=ENST00000503134;v=rs914648291;vf=182168040)[A](https://uswest.ensembl.org/Homo_sapiens/ZMenu/TextSequence?db=core;factorytype=Location;g=ENSG00000237187;r=5:93399495-93595784;t=ENST00000503134;v=rs914648291;v=rs561454399;vf=182168040;vf=93944767)[A](https://uswest.ensembl.org/Homo_sapiens/ZMenu/TextSequence?db=core;factorytype=Location;g=ENSG00000237187;r=5:93399495-93595784;t=ENST00000503134;v=rs914648291;vf=182168040)

[ACCTTTCTA](https://uswest.ensembl.org/Homo_sapiens/ZMenu/TextSequence?db=core;factorytype=Location;g=ENSG00000237187;r=5:93399495-93595784;t=ENST00000503134;v=rs914648291;vf=182168040)TGTAC[A](https://uswest.ensembl.org/Homo_sapiens/ZMenu/TextSequence?db=core;factorytype=Location;g=ENSG00000237187;r=5:93399495-93595784;t=ENST00000503134;v=rs964688146;vf=232223293)GA[T](https://uswest.ensembl.org/Homo_sapiens/ZMenu/TextSequence?db=core;factorytype=Location;g=ENSG00000237187;r=5:93399495-93595784;t=ENST00000503134;v=rs148302385;vf=32690554)TCTA[A](https://uswest.ensembl.org/Homo_sapiens/ZMenu/TextSequence?db=core;factorytype=Location;g=ENSG00000237187;r=5:93399495-93595784;t=ENST00000503134;v=rs1008335395;vf=275883995)ATCGT[A](https://uswest.ensembl.org/Homo_sapiens/ZMenu/TextSequence?db=core;factorytype=Location;g=ENSG00000237187;r=5:93399495-93595784;t=ENST00000503134;v=rs573400409;vf=105847162)CT[C](https://uswest.ensembl.org/Homo_sapiens/ZMenu/TextSequence?db=core;factorytype=Location;g=ENSG00000237187;r=5:93399495-93595784;t=ENST00000503134;v=rs559883842;vf=92379823)CCCATACAGTC[A](https://uswest.ensembl.org/Homo_sapiens/ZMenu/TextSequence?db=core;factorytype=Location;g=ENSG00000237187;r=5:93399495-93595784;t=ENST00000503134;v=rs201941521;vf=50047800)GAGGCA[G](https://uswest.ensembl.org/Homo_sapiens/ZMenu/TextSequence?db=core;factorytype=Location;g=ENSG00000237187;r=5:93399495-93595784;t=ENST00000503134;v=rs115984302;vf=22746835)GGAGTCGAG

G[G](https://uswest.ensembl.org/Homo_sapiens/ZMenu/TextSequence?db=core;factorytype=Location;g=ENSG00000237187;r=5:93399495-93595784;t=ENST00000503134;v=rs1007838806;vf=275387253)CAGAGGCAGCAGG[C](https://uswest.ensembl.org/Homo_sapiens/ZMenu/TextSequence?db=core;factorytype=Location;g=ENSG00000237187;r=5:93399495-93595784;t=ENST00000503134;v=rs557654765;vf=90158965)GG[A](https://uswest.ensembl.org/Homo_sapiens/ZMenu/TextSequence?db=core;factorytype=Location;g=ENSG00000237187;r=5:93399495-93595784;t=ENST00000503134;v=rs1033218584;vf=300774825)GAGAGGGA[G](https://uswest.ensembl.org/Homo_sapiens/ZMenu/TextSequence?db=core;factorytype=Location;g=ENSG00000237187;r=5:93399495-93595784;t=ENST00000503134;v=rs1038613459;vf=306171372)ATAGGGCGAGGGAAGGA[G](https://uswest.ensembl.org/Homo_sapiens/ZMenu/TextSequence?db=core;factorytype=Location;g=ENSG00000237187;r=5:93399495-93595784;t=ENST00000503134;v=rs999965032;vf=267511024)[A](https://uswest.ensembl.org/Homo_sapiens/ZMenu/TextSequence?db=core;factorytype=Location;g=ENSG00000237187;r=5:93399495-93595784;t=ENST00000503134;v=rs902901200;vf=170417303)ACGCGGAG[A](https://uswest.ensembl.org/Homo_sapiens/ZMenu/TextSequence?db=core;factorytype=Location;g=ENSG00000237187;r=5:93399495-93595784;t=ENST00000503134;v=rs746008103;vf=111567738)[G](https://uswest.ensembl.org/Homo_sapiens/ZMenu/TextSequence?db=core;factorytype=Location;g=ENSG00000237187;r=5:93399495-93595784;t=ENST00000503134;v=rs939228268;vf=206755477)AGA

C[G](https://uswest.ensembl.org/Homo_sapiens/ZMenu/TextSequence?db=core;factorytype=Location;g=ENSG00000237187;r=5:93399495-93595784;t=ENST00000503134;v=rs928634813;vf=196158813)AAGG[G](https://uswest.ensembl.org/Homo_sapiens/ZMenu/TextSequence?db=core;factorytype=Location;g=ENSG00000237187;r=5:93399495-93595784;t=ENST00000503134;v=rs949720246;vf=217250736)[A](https://uswest.ensembl.org/Homo_sapiens/ZMenu/TextSequence?db=core;factorytype=Location;g=ENSG00000237187;r=5:93399495-93595784;t=ENST00000503134;v=rs896108669;vf=163622736)GTGGG[A](https://uswest.ensembl.org/Homo_sapiens/ZMenu/TextSequence?db=core;factorytype=Location;g=ENSG00000237187;r=5:93399495-93595784;t=ENST00000503134;v=rs977386702;vf=244925808)GA[C](https://uswest.ensembl.org/Homo_sapiens/ZMenu/TextSequence?db=core;factorytype=Location;g=ENSG00000237187;r=5:93399495-93595784;t=ENST00000503134;v=rs1055981739;vf=323545097)[C](https://uswest.ensembl.org/Homo_sapiens/ZMenu/TextSequence?db=core;factorytype=Location;g=ENSG00000237187;r=5:93399495-93595784;t=ENST00000503134;v=rs937550785;vf=205077466)AGC[G](https://uswest.ensembl.org/Homo_sapiens/ZMenu/TextSequence?db=core;factorytype=Location;g=ENSG00000237187;r=5:93399495-93595784;t=ENST00000503134;v=rs537451942;vf=70029555)ACAGCGAGCGCCAGGGCAC[C](https://uswest.ensembl.org/Homo_sapiens/ZMenu/TextSequence?db=core;factorytype=Location;g=ENSG00000237187;r=5:93399495-93595784;t=ENST00000503134;v=rs984667281;vf=252208573)GCA[G](https://uswest.ensembl.org/Homo_sapiens/ZMenu/TextSequence?db=core;factorytype=Location;g=ENSG00000237187;r=5:93399495-93595784;t=ENST00000503134;v=rs369607410;vf=52173149)GCACACTGGATGCC

CAGTCCGC[C](https://uswest.ensembl.org/Homo_sapiens/ZMenu/TextSequence?db=core;factorytype=Location;g=ENSG00000237187;r=5:93399495-93595784;t=ENST00000503134;v=rs930105913;vf=197630364)GGC[C](https://uswest.ensembl.org/Homo_sapiens/ZMenu/TextSequence?db=core;factorytype=Location;g=ENSG00000237187;r=5:93399495-93595784;t=ENST00000503134;v=rs143221381;vf=28457938)CAGCCGCCCTCGGACCCTGGA[C](https://uswest.ensembl.org/Homo_sapiens/ZMenu/TextSequence?db=core;factorytype=Location;g=ENSG00000237187;r=5:93399495-93595784;t=ENST00000503134;v=rs555146828;vf=87660059)CGTGCGTCGTGGGCAC[C](https://uswest.ensembl.org/Homo_sapiens/ZMenu/TextSequence?db=core;factorytype=Location;g=ENSG00000237187;r=5:93399495-93595784;t=ENST00000503134;v=rs976787436;vf=244326345)AGA[A](https://uswest.ensembl.org/Homo_sapiens/ZMenu/TextSequence?db=core;factorytype=Location;g=ENSG00000237187;r=5:93399495-93595784;t=ENST00000503134;v=rs965362825;vf=232898174)ACGA

GACT[C](https://uswest.ensembl.org/Homo_sapiens/ZMenu/TextSequence?db=core;factorytype=Location;g=ENSG00000237187;r=5:93399495-93595784;t=ENST00000503134;v=rs535108869;vf=67694950)GG[C](https://uswest.ensembl.org/Homo_sapiens/ZMenu/TextSequence?db=core;factorytype=Location;g=ENSG00000237187;r=5:93399495-93595784;t=ENST00000503134;v=rs1018254527;vf=285806085)CAAATCCT[G](https://uswest.ensembl.org/Homo_sapiens/ZMenu/TextSequence?db=core;factorytype=Location;g=ENSG00000237187;r=5:93399495-93595784;t=ENST00000503134;v=rs987030233;vf=254572268)CCT[C](https://uswest.ensembl.org/Homo_sapiens/ZMenu/TextSequence?db=core;factorytype=Location;g=ENSG00000237187;r=5:93399495-93595784;t=ENST00000503134;v=rs187916685;vf=42446105)ACCAG

Mouse AK044036

ATTGTACGCAGATGATGGAGAGGCGGGACATGGGGTGGCAGGGGCTCCTGGCCTTAGCCT

CTCAG[C](https://uswest.ensembl.org/Mus_musculus/ZMenu/TextSequence?db=core;factorytype=Location;g=ENSMUSG00000087143;r=13:78147895-78284658;t=ENSMUST00000123998;v=rs228907730;vf=32382529)CCTAATATTCGTGATCAAGTCTTCCGGTTCGTTTAAAG

 BAD AVG GOOD
*
human   :  97
mouse   :  97
cons    :  95

human   ATTGTACGCAGCTGATGGCGAGGAGGCGACTGGGGGTAGCAGGGGCTCCTTGCCCTCGTCCCTATGCCCAAATAA
mouse   ATTGTACGCAGATGATGGAGAGGCGG-GACATGGGGTGGCAGGGGCTCCTGGCCTTAGCCTCTCAGCCCTAATAT

cons    *********** ****** **** ** ***  ***** ************ *** * * * **  **** **** 


human   ACGCGCAAACGCGCTCCCGTC---TTCTGG
mouse   TCGTGATCAAGTCTTCCGGTTCGTTTAAAG

cons     ** *   * *   *** **    **   *

Exon b

Human hg 19 (exon 4)

GAAATGTTTATATGGTGTCTTAAAATTAATGAATTTTATTTGTTTGCAGGTGGCAGTGGGCCTGCATCACAGGTTGCAGCAGATGTTCTCAATATTTCTATTAAAATTTCCTTATTTCCATATGCAAGAGGAGCCCCAGAGCTGCATCCTTATGGTAGCTACCATGCCGTGGTAGGTGATGTTGTACTTTTCCACATCATCCTTCCTGTGctaacagaggatctgggttagaatctagctgtttgtttttgtaaacaactatggagcactgaaagaagaatgtttcaataatat

Mouse mm9 (exon 3)

ACCATGTTTTTGCAGTGCTTTAAAGCTAACCGAACTTATTTGTTTGCAGGTGGCCATGGAATGGTGTAGCAGATTGCTGCAGATGGTTTCAATAGTTCTGCTGAATGCTCCTTGTTTGTGCCTGCAAGAGGAACTCAAGGCTTTATGTATGGCCATTGCTGTGACCTAGTGTGATGCTCTACTTTTCCACTGAACTCTGCTTGCTCTAACTGAGGACCTTAGCTATAATTTAGCCTTTTCTTTTTGTAAGTAACTACACATAGTTGAGACAAAATTATTTTGATGT

*
 BAD AVG GOOD
*
Human   :  97
Mouse   :  98
cons    :  97

Human   GAAATGTTTATATGGTGTCTTAAAATTAATGAATTTTATTTGTTTGCAGGTGGCAGTGGGCCTGCATCACAGGTT
Mouse   ACCATGTTTTTGCAGTGCTTTAAAGCTAACCGAACTTATTTGTTTGCAGGTGGCCATGGAATGGTGTAGCAGATT

cons       ****** *   ***  *****  ***   *  *******************  ***    *  *  *** **


Human   GCAGCAGATGTTCTCAATATTTCTATTAAAATTTCCTTATTTCCATATGCAAGAGGAGCCCCAGAGCTGCATCCT
Mouse   GCTGCAGATGGTTTCAATAGTTCTGCTGAATGCTCCTTGTTTGTGCCTGCAAGAGGAACTC-AAGGCTTTAT--G

cons    ** ******* * ****** ****  * **   ***** ***     ********** * * *  ***  **   


Human   TATGGTAGCTACCATGCCGTGGTAGGTGATGTTGTACTTTTCCACATCATCCTTCCTGTGCTAACAGAGGATCTG
Mouse   TATGGCCATTGCTGTGACCTAGT--GTGATGCTCTACTTTTCCACTGAACTCTGCTTGCTCTAACTGAGGACCTT

cons    *****    * *  ** * * **  ****** * ***********   *  ** * **  ***** ***** ** 


Human   GGTTAGAATCTAGCTGTTTGTTTTTGTAAACAACTATGGAGCACTGAAAGAAGAATGTTTCAATAATAT
Mouse   AGCTATAATTTAGCCTTTTCTTTTTGTAAGTAACTACACATAGTTGAGACAAAATTATTT---TGATGT

cons     * ** *** ****  *** *********  *****   *    *** * ** * * ***   * ** *

Exon c

>Human exon 5

ATGTAAGCTGCCATTCACTGGGGAGCTTTGGCAATAGAATTGGCTAGATCAGGAAGCCTATGTCAACTATGGAACAACAACTTGCAGCTCATCTCTATTGAT

>Mouse exon 4

ATATTAAGCTGTTGTTGAGGAAGGAGCTTTGGCAATAGAAGAAA[C](http://uswest.ensembl.org/Mus_musculus/ZMenu/TextSequence?db=core;factorytype=Location;g=ENSMUSG00000087143;r=13:78198017-78279391;t=ENSMUST00000145613;v=rs246703049;vf=50139441)CAAGCAGCAGGTT[A](http://uswest.ensembl.org/Mus_musculus/ZMenu/TextSequence?db=core;factorytype=Location;g=ENSMUSG00000087143;r=13:78198017-78279391;t=ENSMUST00000145613;v=rs261922777;vf=65326292)ATTTCCACTGTTGACACAACTGGAAACGGATCCCTACTTTATGCATTGGAGATGCTATGAAGACTACTCTGTCTGATGTTTCCCTA[T](http://uswest.ensembl.org/Mus_musculus/ZMenu/TextSequence?db=core;factorytype=Location;g=ENSMUSG00000087143;r=13:78198017-78279391;t=ENSMUST00000145613;v=rs228043496;vf=31520170)TGGG[A](http://uswest.ensembl.org/Mus_musculus/ZMenu/TextSequence?db=core;factorytype=Location;g=ENSMUSG00000087143;r=13:78198017-78279391;t=ENSMUST00000145613;v=rs251388533;vf=54814939)TAGTGAAGT[A](http://uswest.ensembl.org/Mus_musculus/ZMenu/TextSequence?db=core;factorytype=Location;g=ENSMUSG00000087143;r=13:78198017-78279391;t=ENSMUST00000145613;v=rs213935615;vf=17442632)TGATGCTTCATTGGTTGAGTGTATGCAATCAAAGTAAGAGTGAAAAAGGAAGATCTGAGGAGTGGGCAGATGCCTGCCGGGTAAA[C](http://uswest.ensembl.org/Mus_musculus/ZMenu/TextSequence?db=core;factorytype=Location;g=ENSMUSG00000087143;r=13:78198017-78279391;t=ENSMUST00000145613;v=rs48225489;vf=10226385)TATTTGTGGCATAAACTTGAGGACCCCACAGGAAA[G](http://uswest.ensembl.org/Mus_musculus/ZMenu/TextSequence?db=core;factorytype=Location;g=ENSMUSG00000087143;r=13:78198017-78279391;t=ENSMUST00000145613;v=rs246171734;vf=49609289)CCAGATGTTGTCATGTGCTTC[A](http://uswest.ensembl.org/Mus_musculus/ZMenu/TextSequence?db=core;factorytype=Location;g=ENSMUSG00000087143;r=13:78198017-78279391;t=ENSMUST00000145613;v=rs29546125;vf=2310662)AACCACAATTCTGCTATTGAATGGGGGATTACTGG[T](http://uswest.ensembl.org/Mus_musculus/ZMenu/TextSequence?db=core;factorytype=Location;g=ENSMUSG00000087143;r=13:78198017-78279391;t=ENSMUST00000145613;v=rs235242382;vf=38703562)CAGTGACTAGCCAAAATGGTGAGCTTCATGTGCTGAGAGAACTACCTATCTC[A](http://uswest.ensembl.org/Mus_musculus/ZMenu/TextSequence?db=core;factorytype=Location;g=ENSMUSG00000087143;r=13:78198017-78279391;t=ENSMUST00000145613;v=rs253880528;vf=57301549)AAAAAATAAGGTGAAAGGGCTAGAGAGGTGGCAAAGTAGCACTGACTGATCTCTCAGAGGAGAA[C](http://uswest.ensembl.org/Mus_musculus/ZMenu/TextSequence?db=core;factorytype=Location;g=ENSMUSG00000087143;r=13:78198017-78279391;t=ENSMUST00000145613;v=rs261796563;vf=65200362)GTGG[A](http://uswest.ensembl.org/Mus_musculus/ZMenu/TextSequence?db=core;factorytype=Location;g=ENSMUSG00000087143;r=13:78198017-78279391;t=ENSMUST00000145613;v=rs220911551;vf=24403761)TTCAA[C](http://uswest.ensembl.org/Mus_musculus/ZMenu/TextSequence?db=core;factorytype=Location;g=ENSMUSG00000087143;r=13:78198017-78279391;t=ENSMUST00000145613;v=rs239314243;vf=42766594)TCCTAGCACCCACATAGCAGCCAACAACCATCTGTAACTCCAGTCTCAGGGGAT[T](http://uswest.ensembl.org/Mus_musculus/ZMenu/TextSequence?db=core;factorytype=Location;g=ENSMUSG00000087143;r=13:78198017-78279391;t=ENSMUST00000145613;v=rs257311990;vf=60725599)TAGTGCCTT[C](http://uswest.ensembl.org/Mus_musculus/ZMenu/TextSequence?db=core;factorytype=Location;g=ENSMUSG00000087143;r=13:78198017-78279391;t=ENSMUST00000145613;v=rs221565263;vf=25056041)TTATATCCTCAGTGAACACTGCACATATATTGTGCATAGACATACATTCAGTCAAACAAACACTCAGA[C](http://uswest.ensembl.org/Mus_musculus/ZMenu/TextSequence?db=core;factorytype=Location;g=ENSMUSG00000087143;r=13:78198017-78279391;t=ENSMUST00000145613;v=rs239380901;vf=42833108)ACATAAAAATAAATGAAATCTCAAAATATTTTAAAAAGATAATGTGGAG[G](http://uswest.ensembl.org/Mus_musculus/ZMenu/TextSequence?db=core;factorytype=Location;g=ENSMUSG00000087143;r=13:78198017-78279391;t=ENSMUST00000145613;v=rs260029962;vf=63437702)GTGATTGAGGCCTTATATTAATCTTTA[G](http://uswest.ensembl.org/Mus_musculus/ZMenu/TextSequence?db=core;factorytype=Location;g=ENSMUSG00000087143;r=13:78198017-78279391;t=ENSMUST00000145613;v=rs222038235;vf=25528040)ACTTTACAAAATGAGTTT[T](http://uswest.ensembl.org/Mus_musculus/ZMenu/TextSequence?db=core;factorytype=Location;g=ENSMUSG00000087143;r=13:78198017-78279391;t=ENSMUST00000145613;v=rs226707946;vf=30187514)[A](http://uswest.ensembl.org/Mus_musculus/ZMenu/TextSequence?db=core;factorytype=Location;g=ENSMUSG00000087143;r=13:78198017-78279391;t=ENSMUST00000145613;v=rs222443333;v=rs226707946;vf=25932286;vf=30187514)[C](http://uswest.ensembl.org/Mus_musculus/ZMenu/TextSequence?db=core;factorytype=Location;g=ENSMUSG00000087143;r=13:78198017-78279391;t=ENSMUST00000145613;v=rs222443333;vf=25932286)ACACACACACACAC[ACAG](http://uswest.ensembl.org/Mus_musculus/ZMenu/TextSequence?db=core;factorytype=Location;g=ENSMUSG00000087143;r=13:78198017-78279391;t=ENSMUST00000145613;v=rs253723662;vf=57145056)AA[A](http://uswest.ensembl.org/Mus_musculus/ZMenu/TextSequence?db=core;factorytype=Location;g=ENSMUSG00000087143;r=13:78198017-78279391;t=ENSMUST00000145613;v=rs242258853;vf=45704836)ACACACAAACACACTATATGTACACACACATATATACACTGACACACACACTTGAAAAA[T](http://uswest.ensembl.org/Mus_musculus/ZMenu/TextSequence?db=core;factorytype=Location;g=ENSMUSG00000087143;r=13:78198017-78279391;t=ENSMUST00000145613;v=rs264867240;vf=68264371)TGTTATTG[C](http://uswest.ensembl.org/Mus_musculus/ZMenu/TextSequence?db=core;factorytype=Location;g=ENSMUSG00000087143;r=13:78198017-78279391;t=ENSMUST00000145613;v=rs583162685;vf=75574190)TTAAAA[T](http://uswest.ensembl.org/Mus_musculus/ZMenu/TextSequence?db=core;factorytype=Location;g=ENSMUSG00000087143;r=13:78198017-78279391;t=ENSMUST00000145613;v=rs260547610;vf=63954204)TTTTTTGATAACTTGTCTCAGAATTTTTATTATTGATTTTATTATAATATT[A](http://uswest.ensembl.org/Mus_musculus/ZMenu/TextSequence?db=core;factorytype=Location;g=ENSMUSG00000087143;r=13:78198017-78279391;t=ENSMUST00000145613;v=rs227559833;vf=31037570)ATTTT[T](http://uswest.ensembl.org/Mus_musculus/ZMenu/TextSequence?db=core;factorytype=Location;g=ENSMUSG00000087143;r=13:78198017-78279391;t=ENSMUST00000145613;v=rs52547673;vf=13957714)AGTCTACAGTAAATCTTTATGCTGGAACTAGACTTCAGGATTATTATAAGATA[T](http://uswest.ensembl.org/Mus_musculus/ZMenu/TextSequence?db=core;factorytype=Location;g=ENSMUSG00000087143;r=13:78198017-78279391;t=ENSMUST00000145613;v=rs257386947;vf=60800403)ATTTAATGTATAAATTTGAGTTCATAAT[T](http://uswest.ensembl.org/Mus_musculus/ZMenu/TextSequence?db=core;factorytype=Location;g=ENSMUSG00000087143;r=13:78198017-78279391;t=ENSMUST00000145613;v=rs227925845;vf=31402766)G[T](http://uswest.ensembl.org/Mus_musculus/ZMenu/TextSequence?db=core;factorytype=Location;g=ENSMUSG00000087143;r=13:78198017-78279391;t=ENSMUST00000145613;v=rs246273966;vf=49711295)GAGAATAATTCTTTTATGG[T](http://uswest.ensembl.org/Mus_musculus/ZMenu/TextSequence?db=core;factorytype=Location;g=ENSMUSG00000087143;r=13:78198017-78279391;t=ENSMUST00000145613;v=rs217197400;vf=20697411)TGTGGTAATAAAGTATCATTATCTTCTTAGATCTAATGTAAGATTCAAACTCTGACCCACTAACTT[C](http://uswest.ensembl.org/Mus_musculus/ZMenu/TextSequence?db=core;factorytype=Location;g=ENSMUSG00000087143;r=13:78198017-78279391;t=ENSMUST00000145613;v=rs235307918;vf=38768966)[C](http://uswest.ensembl.org/Mus_musculus/ZMenu/TextSequence?db=core;factorytype=Location;g=ENSMUSG00000087143;r=13:78198017-78279391;t=ENSMUST00000145613;v=rs249524594;vf=52954969)AAGTCCCTTCTTAAAAGTGCTGTATTAAACTGCATGAA[G](http://uswest.ensembl.org/Mus_musculus/ZMenu/TextSequence?db=core;factorytype=Location;g=ENSMUSG00000087143;r=13:78198017-78279391;t=ENSMUST00000145613;v=rs587339362;vf=79738296)CTTTTT[TT](http://uswest.ensembl.org/Mus_musculus/ZMenu/TextSequence?db=core;factorytype=Location;g=ENSMUSG00000087143;r=13:78198017-78279391;t=ENSMUST00000123998;v=rs232029212;vf=35497270)CCCTTTTTTCTTTTTCAAGTCAG[G](http://uswest.ensembl.org/Mus_musculus/ZMenu/TextSequence?db=core;factorytype=Location;g=ENSMUSG00000087143;r=13:78198017-78279391;t=ENSMUST00000123998;v=rs212739598;vf=16249159)GTTATTTTGTGTAGTCCTAATTGTCC[C](http://uswest.ensembl.org/Mus_musculus/ZMenu/TextSequence?db=core;factorytype=Location;g=ENSMUSG00000087143;r=13:78198017-78279391;t=ENSMUST00000123998;v=rs50848511;vf=12521833)GGAACAATGGTTGCCTGTGAGCCCCAGCATCTGTAATAGTCAGGAT[C](http://uswest.ensembl.org/Mus_musculus/ZMenu/TextSequence?db=core;factorytype=Location;g=ENSMUSG00000087143;r=13:78198017-78279391;t=ENSMUST00000123998;v=rs262168178;vf=65571202)TGGCAGAG[C](http://uswest.ensembl.org/Mus_musculus/ZMenu/TextSequence?db=core;factorytype=Location;g=ENSMUSG00000087143;r=13:78198017-78279391;t=ENSMUST00000123998;v=rs580490997;vf=72910687)CT[C](http://uswest.ensembl.org/Mus_musculus/ZMenu/TextSequence?db=core;factorytype=Location;g=ENSMUSG00000087143;r=13:78198017-78279391;t=ENSMUST00000123998;v=rs221637667;vf=25128280)TCAGGAGACACTGTATCAGGCTCCTGTCAGCAAGCACTTCTTGGCATCAGAAATAGTGTCT[C](http://uswest.ensembl.org/Mus_musculus/ZMenu/TextSequence?db=core;factorytype=Location;g=ENSMUSG00000087143;r=13:78198017-78279391;t=ENSMUST00000123998;v=rs233086461;vf=36552318)GGTTTGGTGTTTGCAT[A](http://uswest.ensembl.org/Mus_musculus/ZMenu/TextSequence?db=core;factorytype=Location;g=ENSMUSG00000087143;r=13:78198017-78279391;t=ENSMUST00000123998;v=rs253379101;vf=56801264)TGGGATGGAT[C](http://uswest.ensembl.org/Mus_musculus/ZMenu/TextSequence?db=core;factorytype=Location;g=ENSMUSG00000087143;r=13:78198017-78279391;t=ENSMUST00000123998;v=rs222093478;vf=25583165)CCCAGGTG[A](http://uswest.ensembl.org/Mus_musculus/ZMenu/TextSequence?db=core;factorytype=Location;g=ENSMUSG00000087143;r=13:78198017-78279391;t=ENSMUST00000123998;v=rs29224394;vf=2000449)GGCAGCCTCTGGAAGGTCTTTTCTTTCAGTCTCTGCTCCACTTTTTGTC[C](http://uswest.ensembl.org/Mus_musculus/ZMenu/TextSequence?db=core;factorytype=Location;g=ENSMUSG00000087143;r=13:78198017-78279391;t=ENSMUST00000123998;v=rs255523569;vf=58941045)CTGTATTTCCCTCAGAAAGGAGCAATTCTGGGTTAAA[AT](http://uswest.ensembl.org/Mus_musculus/ZMenu/TextSequence?db=core;factorytype=Location;g=ENSMUSG00000087143;r=13:78198017-78279391;t=ENSMUST00000123998;v=rs216843333;vf=20344092)TTTTGAGATGGGTGGGTGG[T](http://uswest.ensembl.org/Mus_musculus/ZMenu/TextSequence?db=core;factorytype=Location;g=ENSMUSG00000087143;r=13:78198017-78279391;t=ENSMUST00000123998;v=rs220098921;vf=23592848)[C](http://uswest.ensembl.org/Mus_musculus/ZMenu/TextSequence?db=core;factorytype=Location;g=ENSMUSG00000087143;r=13:78198017-78279391;t=ENSMUST00000123998;v=rs244060308;vf=47502398)CCATCCCTCAAGTGGGATGCCATGCCTAACCTCGAGATATGGTCTCAACAGGTTTTCATTCCCCTTTGTGGG[GCAT](http://uswest.ensembl.org/Mus_musculus/ZMenu/TextSequence?db=core;factorytype=Location;g=ENSMUSG00000087143;r=13:78198017-78279391;t=ENSMUST00000123998;v=rs220382413;vf=23875731)TTCAGCTAATGTCATC[C](http://uswest.ensembl.org/Mus_musculus/ZMenu/TextSequence?db=core;factorytype=Location;g=ENSMUSG00000087143;r=13:78198017-78279391;t=ENSMUST00000123998;v=rs257495467;vf=60908691)CCATGGGGTCCTGGGAGGCTCTTGCTTTCCTGGCATT[T](http://uswest.ensembl.org/Mus_musculus/ZMenu/TextSequence?db=core;factorytype=Location;g=ENSMUSG00000087143;r=13:78198017-78279391;t=ENSMUST00000123998;v=rs228029784;vf=31506487)GGGACTTTCTGGTT[G](http://uswest.ensembl.org/Mus_musculus/ZMenu/TextSequence?db=core;factorytype=Location;g=ENSMUSG00000087143;r=13:78198017-78279391;t=ENSMUST00000123998;v=rs29681704;vf=2442160)CTACCCCCAGTTCTGTGAGGAGAGGGTGTGGCAG[T](http://uswest.ensembl.org/Mus_musculus/ZMenu/TextSequence?db=core;factorytype=Location;g=ENSMUSG00000087143;r=13:78198017-78279391;t=ENSMUST00000123998;v=rs261097695;vf=64503012)AGTCCCAA[T](http://uswest.ensembl.org/Mus_musculus/ZMenu/TextSequence?db=core;factorytype=Location;g=ENSMUSG00000087143;r=13:78198017-78279391;t=ENSMUST00000123998;v=rs584081683;vf=76490442)ATGGC[G](http://uswest.ensembl.org/Mus_musculus/ZMenu/TextSequence?db=core;factorytype=Location;g=ENSMUSG00000087143;r=13:78198017-78279391;t=ENSMUST00000123998;v=rs232877517;vf=36343778)CC[T](http://uswest.ensembl.org/Mus_musculus/ZMenu/TextSequence?db=core;factorytype=Location;g=ENSMUSG00000087143;r=13:78198017-78279391;t=ENSMUST00000123998;v=rs586755840;vf=79156488)[G](http://uswest.ensembl.org/Mus_musculus/ZMenu/TextSequence?db=core;factorytype=Location;g=ENSMUSG00000087143;r=13:78198017-78279391;t=ENSMUST00000123998;v=rs581402126;vf=73818961)GGACTG[T](http://uswest.ensembl.org/Mus_musculus/ZMenu/TextSequence?db=core;factorytype=Location;g=ENSMUSG00000087143;r=13:78198017-78279391;t=ENSMUST00000123998;v=rs579517042;vf=71939618)[G](http://uswest.ensembl.org/Mus_musculus/ZMenu/TextSequence?db=core;factorytype=Location;g=ENSMUSG00000087143;r=13:78198017-78279391;t=ENSMUST00000123998;v=rs587122155;vf=79521742)ACTAAGTCTTATGACTT[G](http://uswest.ensembl.org/Mus_musculus/ZMenu/TextSequence?db=core;factorytype=Location;g=ENSMUSG00000087143;r=13:78198017-78279391;t=ENSMUST00000123998;v=rs580628127;vf=73047406)CACCTGA[C](http://uswest.ensembl.org/Mus_musculus/ZMenu/TextSequence?db=core;factorytype=Location;g=ENSMUSG00000087143;r=13:78198017-78279391;t=ENSMUST00000123998;v=rs51665639;vf=13241982)TTCCTCATACA[C](http://uswest.ensembl.org/Mus_musculus/ZMenu/TextSequence?db=core;factorytype=Location;g=ENSMUSG00000087143;r=13:78198017-78279391;t=ENSMUST00000123998;v=rs584324555;vf=76732577)CTGAAAATAAGCC[A](http://uswest.ensembl.org/Mus_musculus/ZMenu/TextSequence?db=core;factorytype=Location;g=ENSMUSG00000087143;r=13:78198017-78279391;t=ENSMUST00000123998;v=rs47547768;vf=9626788)[T](http://uswest.ensembl.org/Mus_musculus/ZMenu/TextSequence?db=core;factorytype=Location;g=ENSMUSG00000087143;r=13:78198017-78279391;t=ENSMUST00000123998;v=rs51162441;v=rs578545908;vf=12798270;vf=70971407)GAC[C](http://uswest.ensembl.org/Mus_musculus/ZMenu/TextSequence?db=core;factorytype=Location;g=ENSMUSG00000087143;r=13:78198017-78279391;t=ENSMUST00000123998;v=rs585251417;vf=77656665)AT[C](http://uswest.ensembl.org/Mus_musculus/ZMenu/TextSequence?db=core;factorytype=Location;g=ENSMUSG00000087143;r=13:78198017-78279391;t=ENSMUST00000123998;v=rs578879798;vf=71304277)GTGAGAACTGTGCAGGTGCACCATGATGCTGGCGGTGTAAACAAG[T](http://uswest.ensembl.org/Mus_musculus/ZMenu/TextSequence?db=core;factorytype=Location;g=ENSMUSG00000087143;r=13:78198017-78279391;t=ENSMUST00000123998;v=rs582563044;vf=74976348)CCATATTTGGTGGAGATG[T](http://uswest.ensembl.org/Mus_musculus/ZMenu/TextSequence?db=core;factorytype=Location;g=ENSMUSG00000087143;r=13:78198017-78279391;t=ENSMUST00000123998;v=rs585300272;vf=77705356)GCCCCTGC[C](http://uswest.ensembl.org/Mus_musculus/ZMenu/TextSequence?db=core;factorytype=Location;g=ENSMUSG00000087143;r=13:78198017-78279391;t=ENSMUST00000123998;v=rs579966848;vf=72388129)G[C](http://uswest.ensembl.org/Mus_musculus/ZMenu/TextSequence?db=core;factorytype=Location;g=ENSMUSG00000087143;r=13:78198017-78279391;t=ENSMUST00000123998;v=rs582395415;vf=74809183)CCT[A](http://uswest.ensembl.org/Mus_musculus/ZMenu/TextSequence?db=core;factorytype=Location;g=ENSMUSG00000087143;r=13:78198017-78279391;t=ENSMUST00000123998;v=rs585488446;vf=77892938)ATTGGCTGAAGCCA[C](http://uswest.ensembl.org/Mus_musculus/ZMenu/TextSequence?db=core;factorytype=Location;g=ENSMUSG00000087143;r=13:78198017-78279391;t=ENSMUST00000123998;v=rs579731881;vf=72153811)[G](http://uswest.ensembl.org/Mus_musculus/ZMenu/TextSequence?db=core;factorytype=Location;g=ENSMUSG00000087143;r=13:78198017-78279391;t=ENSMUST00000123998;v=rs583717858;vf=76127725)TGCCTGGTGAGGTGACATGGCCTGCC[G](http://uswest.ensembl.org/Mus_musculus/ZMenu/TextSequence?db=core;factorytype=Location;g=ENSMUSG00000087143;r=13:78198017-78279391;t=ENSMUST00000123998;v=rs248539984;vf=51972513)TGAGTGGATGGGGGCTGAGAGTATATAAGAGTGAGAGGCC[T](http://uswest.ensembl.org/Mus_musculus/ZMenu/TextSequence?db=core;factorytype=Location;g=ENSMUSG00000087143;r=13:78198017-78279391;t=ENSMUST00000123998;v=rs587498996;vf=79897426)GGGGTTCA[G](http://uswest.ensembl.org/Mus_musculus/ZMenu/TextSequence?db=core;factorytype=Location;g=ENSMUSG00000087143;r=13:78198017-78279391;t=ENSMUST00000123998;v=rs580004183;vf=72425342)GGGGG[A](http://uswest.ensembl.org/Mus_musculus/ZMenu/TextSequence?db=core;factorytype=Location;g=ENSMUSG00000087143;r=13:78198017-78279391;t=ENSMUST00000123998;v=rs212276841;vf=15787330)AGAGATGAAGAGGGAGAGAGA[TG](http://uswest.ensembl.org/Mus_musculus/ZMenu/TextSequence?db=core;factorytype=Location;g=ENSMUSG00000087143;r=13:78198017-78279391;t=ENSMUST00000123998;v=rs46663658;vf=8842576)AAGACTGAAGATTGCTGAATAAACT[A](http://uswest.ensembl.org/Mus_musculus/ZMenu/TextSequence?db=core;factorytype=Location;g=ENSMUSG00000087143;r=13:78198017-78279391;t=ENSMUST00000123998;v=rs52584571;vf=13985552)CTGTTAGAA[G](http://uswest.ensembl.org/Mus_musculus/ZMenu/TextSequence?db=core;factorytype=Location;g=ENSMUSG00000087143;r=13:78198017-78279391;t=ENSMUST00000123998;v=rs583673541;vf=76083523)

 BAD AVG GOOD
*
Mouse   :  93
Human   :  94
cons    :  88

Mouse   ATATTAAGCTGTTGTTGAGGAAGGAGCTTTGGCAATAGAAGAAACCA-AGCAGCAGGTTAATTTCCACTGTTGA-
Human   ATG-TAAGCTGCCATTCACTGGGGAGCTTTGGCAATAGAATTGGCTAGATCAGGAAGCCTATGTCAACTATGGAA

cons    **  *******   ** *    ******************    * * * *** * *   ** ** *** * ** 


Mouse   C-ACAACTGGAAACGGATCCCTACTTTATGCAT
Human   CAACAACTTGCAGCTCAT-----CTCTATTGAT

cons    * ****** * * *  **     ** ***  **

Sequences used for discover microhomology (Figure S5C)

**For exon2:**

>mm9_dna range=chr13:78370430-78370737 5'pad=0 3'pad=0 strand=+ repeatMasking=none

ACCATGTTTTTGCAGTGCTTTAAAGCTAACCGAACTTATTTGTTTGCAGG TGGCCATGGAATGGTGTAGCAGATTGCTGCAGATGGTTTCAATAGTTCTG CTGAATGCTCCTTGTTTGTGCCTGCAAGAGGAACTCAAGGCTTTATGTAT GGCCATTGCTGTGACCTAGTGTGATGCTCTACTTTTCCACTGAACTCTGC TTGCTCTAACTGAGGACCTTAGCTATAATTTAGCCTTTTCTTTTTGTAAG TAACTACACATAGTTGAGACAAAATTATTTTGATGTTTAAAAAACAATTT TATTAACC

>rn4_dna range=chr2:5856639-5856957 5'pad=0 3'pad=0 strand=+ repeatMasking=none

ACCATGTTTTTACAGTGCTTTAGAGCTAACAGAGTTTATTTGTTTCCAGG TGGCCATGCGATGGCATCATAGGTTGCTGCAGATGGTTTCAATGGTTCTG TTGGATGTTCTTCATCTGTGCCTGCAAGAGGCGCCCAAGGCTTTATGTAT GGCCATTGCTATGACCTAGTGTGATGCTCTACTTTCCCGCTGAACTTTAC TTGCTAGAACTGAGGAGCTTGGCTATAATTTAGCCTTTTCTTTTTGTAAG TAGCTATGCATAGCTGAGACAAAATTATTTTGCTGTTTAAAAAACAATTT TATTGGTTTTTTTAGTCAG

>bosTau3_dna range=chr7:83221094-83221909 5'pad=300 3'pad=300 strand=- repeatMasking=none

CCTTCTCTGACAGTTTAGTCTAGTGCCTTAGAATTAACAGTTTATTTGTTTACAGGTGGCAGTAGACCCCCATCACAGGTGGTAGCAAATTTTCTCAACATTCCTATTGAAATTTCCTTATCTCCATTTGCAGGAGGAGCCCCAGAGCCTCATCCCTGTGGTAGCTAACTTGTTCTGGTATGATGCTGTGCTCTTCTACTTCATGCTTCCTGTTCTAGCAGAGGACCTGGGTTGGAATTTAGCTGTTTATTATTGCAGGAACTATGTAGAAGTGGAAGTGGGGAATGTTTCAATAATACTGATATTATGATTTCTTTGAGATTGTGTACTATGCCTTGTTATTTTTATACTTGATACGGAACCCCGTATCAACCCCTCCAAATAGACTCAGAGGCTTCCCTGGTGGCTCAGAATCTGTTTGCAATGCAGGAGACCCAGGTTTGATCCCTGGGTCGGGAAGACACCGTGGAGAAGGAAATGGCAACCCACTCTAGTATTCTTGCCTGGAGAATTCCACAGACAGAGGATCCTGGCGGGCT

>hg18_dna range=chr5:92916188-92916506 5'pad=0 3'pad=0 strand=- repeatMasking=none

GAAATGTTTATATGGTGTCTTAAAATTAATGAATTTTATTTGTTTGCAGG TGGCAGTGGGCCTGCATCACAGGTTGCAGCAGATGTTCTCAATATTTCTA TTAAAATTTCCTTATTTCCATATGCAAGAGGAGCCCCAGAGCTGCATCCT TATGGTAGCTACCATGCCGTGGTAGGTGATGTTGTACTTTTCCACATCAT CCTTCCTGTGCTAACAGAGGATCTGGGTTAGAATCTAGCTGTTTGTTTTT GTAAACAACTATGGAGCACTGAAAGAAGAATGTTTCAATAATATCTAAAT

TGTGATTTCTTTGAGGTTA

>panTro2_dna range=chr5:22003150-22003468 5'pad=0 3'pad=0 strand=+ repeatMasking=none

GAAATGTTTATATGGTGTCTTAAAATTAATGAATTTTATTTGTTTGCAGGTGGCAGTGGGCCTGCATCACAGGTTGCAGCAGATGTTCTCAATATTTCTATTAAAATTTCCTTATTTCCATATGCAAGAGGAGCCCCAGAGCTGCATCCTTATGGTAGCTACCATGCCGTGGTAGGTGATGCTGTACTTTTCCACATCATCCTTCCTGTGCTAACAGAGGATCTGGGTTAGAATCTAGCTGTTTGTTTTTGTAAACAACTATGGAGCACTGAAAGAAGAATGTTTCAATAATATCTAAATTGTGATTTCTTTGGGGTTA

>canFam2_dna range=chr3:18628033-18628400 5'pad=0 3'pad=0 strand=+ repeatMasking=none

GAGACATTTGTGTAGTGCCTTTATATTAACAAAGTTTATTTGTTTACAGGTGGCAGTGGGCAACATCATGGGTTGTAGCAGATGTTCTCAACATTTCTTTTGAAACTTCTTTATATTGCCTTGCAAGAGGAGCCCTAGAGCCTCATCCCTGGGGTAGCTACTATGCTCTGGTATGATGCTGTGCTGTTCTATTTCATCCTTCCTGTTCTAGCAGAGGATCTAGGTTAGAATTTAGTGGTTTCTTATTGCAGACAATTATGTAGAACTGGAAGTGGAATGTTTGAATTATACTGATATTGTGATTTCTTTGAGATTGTTTATTATGCCTTATTATTCTTATACTTGTTCACAGAACCCCCAAATAGTGA

**For exon3:**

>mm9_dna range=chr13:78373352-78376424 5'pad=300 3'pad=300 strand=+ repeatMasking=none

TAGGAACACATTATTACACAACTTCTTTACTAAGGAGAGTGAATAAGTAG

CTAAAAATTAAAGTACTTTTTCTGCATGAAAAGTGGGAATATTTTTCAGA

ATTCTGGGAAATGTCAAAGTAATTTTGGTTGTAGTCATATTGCATTTAAT

GCTATTGAGAATATTGCTAACATTAACAAAAATATTCTGTAATTTCCACA

GTTATTGCTATTGGTGACAAGGGATGTGAGTTGGCTTGCATCCATGCTTT

GAGCATCACCATTAGACACATCTGGTTCCACATGTCAGAAACAATCTTTC

CTTTAAGACAGTTGAGAAAATTTTCAGTTGGGGGTAGTGGTTAGGGTTGA

TTATTCTATATTTTAATCAATGATATTTTAGAGTATAACAAATGTTCCAG

AGTAAACACAAGTGTAAAATTTTATGTATAAGACAATTTTTAAACATTTC

TTTGATTTTTAAAAAAGTTTATCAGAATATAGTGGTTTGGAACCAAAGGA

AAATTTTATTCTATAATAATCATTTTATCTTTTTGTTTTTCCCTGTGACA

TTGGCAACATCCACTTTACAGATATTAAGCTGTTGTTGAGGAAGGAGCTT

TGGCAATAGAAGAAACCAAGCAGCAGGTTAATTTCCACTGTTGACACAAC

TGGAAACGGATCCCTACTTTATGCATTGGAGATGCTATGAAGACTACTCT

GTCTGATGTTTCCCTATTGGGATAGTGAAGTATGATGCTTCATTGGTTGA

GTGTATGCAATCAAAGTAAGAGTGAAAAAGGAAGATCTGAGGAGTGGGCA

GATGCCTGCCGGGTAAACTATTTGTGGCATAAACTTGAGGACCCCACAGG

AAAGCCAGATGTTGTCATGTGCTTCAAACCACAATTCTGCTATTGAATGG

GGGATTACTGGTCAGTGACTAGCCAAAATGGTGAGCTTCATGTGCTGAGA

GAACTACCTATCTCAAAAAAATAAGGTGAAAGGGCTAGAGAGGTGGCAAA

GTAGCACTGACTGATCTCTCAGAGGAGAACGTGGATTCAACTCCTAGCAC

CCACATAGCAGCCAACAACCATCTGTAACTCCAGTCTCAGGGGATTTAGT

GCCTTCTTATATCCTCAGTGAACACTGCACATATATTGTGCATAGACATA

CATTCAGTCAAACAAACACTCAGACACATAAAAATAAATGAAATCTCAAA

ATATTTTAAAAAGATAATGTGGAGGGTGATTGAGGCCTTATATTAATCTT

TAGACTTTACAAAATGAGTTTTACACACACACACACACACAGAAAACACA

CAAACACACTATATGTACACACACATATATACACTGACACACACACTTGA

AAAATTGTTATTGCTTAAAATTTTTTTGATAACTTGTCTCAGAATTTTTA

TTATTGATTTTATTATAATATTAATTTTTAGTCTACAGTAAATCTTTATG

CTGGAACTAGACTTCAGGATTATTATAAGATATATTTAATGTATAAATTT

GAGTTCATAATTGTGAGAATAATTCTTTTATGGTTGTGGTAATAAAGTAT

CATTATCTTCTTAGATCTAATGTAAGATTCAAACTCTGACCCACTAACTT

CCAAGTCCCTTCTTAAAAGTGCTGTATTAAACTGCATGAAGCTTTTTTTC

CCTTTTTTCTTTTTCAAGTCAGGGTTATTTTGTGTAGTCCTAATTGTCCC

GGAACAATGGTTGCCTGTGAGCCCCAGCATCTGTAATAGTCAGGATCTGG

CAGAGCCTCTCAGGAGACACTGTATCAGGCTCCTGTCAGCAAGCACTTCT

TGGCATCAGAAATAGTGTCTCGGTTTGGTGTTTGCATATGGGATGGATCC

CCAGGTGAGGCAGCCTCTGGAAGGTCTTTTCTTTCAGTCTCTGCTCCACT

TTTTGTCCCTGTATTTCCCTCAGAAAGGAGCAATTCTGGGTTAAAATTTT

TGAGATGGGTGGGTGGTCCCATCCCTCAAGTGGGATGCCATGCCTAACCT

CGAGATATGGTCTCAACAGGTTTTCATTCCCCTTTGTGGGGCATTTCAGC

TAATGTCATCCCCATGGGGTCCTGGGAGGCTCTTGCTTTCCTGGCATTTG

GGACTTTCTGGTTGCTACCCCCAGTTCTGTGAGGAGAGGGTGTGGCAGTA

GTCCCAATATGGCGCCTGGGACTGTGACTAAGTCTTATGACTTGCACCTG

ACTTCCTCATACACCTGAAAATAAGCCATGACCATCGTGAGAACTGTGCA

GGTGCACCATGATGCTGGCGGTGTAAACAAGTCCATATTTGGTGGAGATG

TGCCCCTGCCGCCCTAATTGGCTGAAGCCACGTGCCTGGTGAGGTGACAT

GGCCTGCCGTGAGTGGATGGGGGCTGAGAGTATATAAGAGTGAGAGGCCT

GGGGTTCAGGGGGGAAGAGATGAAGAGGGAGAGAGATGAAGACTGAAGAT

TGCTGAATAAACTACTGTTAGAAGAACTGGTGGTCGTGTTGTTCTTGCTG

GTAGAGAGTAGATGCAACAAATGGTGGCCCATATGGGGAACCAATTCCCC

CACCAATGAGTTCAGAACTTTCAGCAGTCAGTGTTTGCTGGCAGGGTAAG

TTCACGGTAAGTGAAACTTATGACCCCAGGAGTTTGGGAAGGACCTCAGA

TAAAACAGAGGCAAGTTTATACTTGCTAGGAAGCAGGCATAAAGTAAAAG

TGAATTGGGAAGGACCTGGGGTAAAACAGAGGCGAATATAAAGTTGCCAG

GAAACAGGAACAAAGTAAAAGTGAAACAATGGGAGCTTCCTCATTGCGTC

CTATTTCTTCAGCTCTTCAGGAGCTCCTTAAGTGTAAAGGTTTAAAAATT

CAAAGAAAAACCATAGAAAAGTTTCTTGATGAGTGCGATACCGTTGCGCC

TTGGTTCGCTGTCTCAGGCAACCTCACAGTCGCTTGCTGGGAGGAGCTAG

GTAGGAACTTAGATGTTGCCTGGGAGCAGGGGATCTTGGAGGGAGGCATG

AAGGCAGTGTGGAGGATAGTAAGGAGCTGTTTGGATGATGAATGCTGTTG

CAGGGTGCTAGAAGCTAGCCAGT

>hg18_dna range=chr5:92914576-92915904 5'pad=300 3'pad=300 strand=- repeatMasking=none

AGTAGAACTATTTTATTGTAAAACCTCGGTATTAACAAGAATGAACACTGTAGCTAGTAACTAAAATATTTTTTCTTCATAAAAAATGGAAGTGTTTTTCAGAATTCTAGGAAATGTCAAAGTAATTTTGGCTGTAGTCATATTACATTTAATGGTATAGAGAATATTTCTAACATTAACAACAATATTCTGTAATTTCCTCAGTTATTGCTACTGGTGATGAGGGATATAAAGTTGGCATGCTTTCATGTTTTGAGCATCATCATTATATACTTCTGATTTTGCAAGTCAGAAACAATCTTCGTTTTAGTTAAGATAGCTGAGAACATTCTGAGGCAGGGGGTCAGGCTTTCATTATTTGCATATTTAGGTAAGTAATATATAGACTACAATGAATTGATCAGAGTAACTACATATATCATTTTTAATGAAAGAATCATACAGTATTTATACATTTCTGTGACTTTTTAAAAGTTTATCAGAATATGGTAGTTTGGGACCAAGGGCATAGGTTTTATTTCTGTATAGATCATTTTATTTTGTTATTTTCTGTGACCACTGGTAATAATCATCTTACAGATGTAAGCTGCCATTCACTGGGGAGCTTTGGCAATAGAATTGGCTAGATCAGGAAGCCTATGTCAACTATGGAACAACAACTTGCAGCTCATCTCTATTGATGGTGAGTCAGTGTCATCTTAATACATGAAGGATCATGTTAATGTTATTTTATCTTCGCAGCATTATCAAACAACAGTTTGTCTTAAACTTTGTTTTATGAGACATTATTGGAATGTTGAACCATGATGCTTCATTACTTGAACCCAAAGGACAACAAAGAAATCAGCACAACAGTGAAAAATAAAGGAAGTTATAATTTGTTATCTGTGAATATACTTAAAATTTGTTTGAGTAGCTTCTGTCATCTCCATTCTCATTCCTTATAACACATGCCAGCTAACTTGTAATTCATGACTCTTATTTGAATACATACTGATTAGAAAAGAGTGATATTGATCACATATGTTGTATACTTATTTTAAAAATATATTACTAGGCCAGGTGTGGTAGCTTATGCCTGTAATCCCAGCACTTTGGGAGGCCAAGGCGAGATCACTTGAGGCCAGAAGTTCGAAACCAGCCTGGCCAACATGTTGAAATCCTGTCTGTACTAAAAACACTTCACTTGGGAAGCTGAGGCACGAGAATTGCTTGAATCTGGGAGGCAGGGGTCGCAGTGAGGTGAGATCACGCCACTGCACTCCGGCCTGGGTGACAGAGCAAGACTGTGTCTCCAAAAAAAAAAAAAAAAAAAAGTGTATTACTGAT

>bosTau3_dna range=chr7:83219031-83221079 5'pad=300 3'pad=300 strand=- repeatMasking=none

TGCACAGGGTCGGACATGACTGAGCGACTAACACTGCTCATCATAGGTACTTAGCATCATGCTTTTGTTTAAATTGTATCCAGTTGTTAGCTGCTGCTTACTCATTTTTTTCTTTTTTTTTATTAAATTTAAAAAATTTTAATTTTTTGGCTACTTTGGCCATACTACGTGGCTTGTGGGATCTTAATTCCCTGACCAGGGAGCAAACCCACGACCCCTGCATTGGAAGCACAAAGTCTTCACCACTGGACCTCCAGGGAAGTTCTGCTTCTTACTGACTTTTAACAAATATTAATCTCTTACTTCCTTAAATAACAAAGAAGCCCATACATTAATTTTACTTTTACTATAAAGTATATCTTCAGCAATGTTTAGCTAGCTTCCTCAACTCTTATGAAGGAGATTCATCTGGCAACTGATTAAATTCCGTATCAGGAATATTTAATTACAATACCTCTGTTCTGCATTAACATGAATGAACACTGAAGCTAGTAACGAAAGTACTTTTTCTTCATGAAAAGTAGAAGTATTTTTCAGGATTCTGGGAAATATTGAAATAATTTTGGTTGTAGTCATACTGTTTTTAATGCTATTGAGAATATTGCTAACATTAACAAAAATATTCTGTAATTTCCTCAGTTATTGCTACTGGTGATGAGGGATGTAAAGTTGGCATGCTTTCATGTTTTGAGCATTTTCATTATATACTTCGGGTTTTACATGTCAGATGCAATTTTTGTTGTAGTTAAGATGGCTGAGAACATTTTTACTTAGGGATAGAGGTCAGGCTGTGGTTATTTGTATTTTTAGGTCAGTGATATATAGAATACAATTAATGTATCAGAGTAAATGCATATGCAATTTTGTTTTTAAGTATTATACAGTACTTATATATTTTTGTGACTTCATTTTTAGTTCATCAAAATATAGTGGTTGGGACCAAGGCATAGGTTTCATTTCTATATTGATAATTTTATTTTGTTACTTTCTGTGACTATTGGTAACACTTATCTTGCAGATACAAGCTCTTATTCACCAGGGATCTTTGGCAAGAGAATGTCTGTATCAGAAAGTCTATCTTCACTGTGGAAAATCAGCTTGAAGCTCGTCTTTGTTGATGGTGAGTCAGTGTCATCTTCATACAGGAAAGATCATACTAGCATTATTTTATATTTTCAGTGTTATCAAACAACTATTTGGTTTAAACTTTTTTTTGAGACAGTATTTTGGGGGTGACAGAGGCTGAGATGGTTGAATGACATAACCGACTCAAGGGACTAGAAAGTGAAAGTCGCTCAGTCATGTAGGACTCTGTGACTCCATGGACTATACAATCCATGGAATTCTCCAGGCCAGAATGCTGGAGTGGGGAGCTGTTCCCTTCTCCAGGGGATCTTCCCAATCCAAGGATCGAACCCAGGTCTCCTGCATCGCAGGTGGATTCTTTACCAGCTGAGCCACCAGGGAAGCCCCAATGGATTTGAGTTTGAGCAAACTCCTGGAGATGGTGAAGGACAGGGAAGCCTAGCATGCTGCAGTCCATGAAATTGCAGAGCTGGACATGACTGAGTGACTAAACAACAACATTAGAATGTTAAACCATAGTGCTTCATTACTTGTGTCTAAAGGATTGCATAAAATTGGAATGGAAGTAATAATTTTCTATTTATGAATATACCTAATAAAAGTAGTTTGATTACCTTCTTTCTCTCACCTCTGTTCTTGTTTCTATATAACACATGGTAACACGACTGAAGCGACTTAGCAGCAGCAGCAGCAGCTGACTTATAATTCATGACTTTTATTTGAATAAATATGGGTAGAATAGAACCACCTGTATTACATGCCTATTTTAAAATATATTACTGAGGTTTAGGAAATTTCAGAATAATTGCAATATAATTGTAAAGATTTTTTTCCATTTTCAAATATATAGATTATGATTTTTCAATGGGTACCTGATGTTAATGCTTGCCGCATGTTTTCTATCTCAGAAACACAGTGAATGACTACATATGTATGTTTAATATTATCACAAGAATTAGTAGT

>panTro2_dna range=chr5:22003244-22004986 5'pad=300 3'pad=300 strand=+ repeatMasking=none

TTTCTATTAAAATTTCCTTATTTCCATATGCAAGAGGAGCCCCAGAGCTG

CATCCTTATGGTAGCTACCATGCCGTGGTAGGTGATGCTGTACTTTTCCA

CATCATCCTTCCTGTGCTAACAGAGGATCTGGGTTAGAATCTAGCTGTTT

GTTTTTGTAAACAACTATGGAGCACTGAAAGAAGAATGTTTCAATAATAT

CTAAATTGTGATTTCTTTGGGGTTATGTACTATGCCTTGTTATTTTTATA

CTTGTTCACAGAACCTCTAAATAGTGCCTTGCTCATCATAGGCACTTAAC

ATTGTGATTTTGTTTTACTTATATCTAGTTTTAATGGCTTATTAATTTTT

AAGTTGTTAATCTAATTTCTTATTCTTTTAAATAATAAATAAGTATGTAA

CATTAATTTTACTTACAAACAATGTCTTCCAGTAATATTTTATCAGCTTC

TCAGCTTTATGAAGGAGATTCAACTAGAAACTGATAAAATTCCATATCAG

GAGTCAGTAGAACTATTTTATTGTAAAACCTCGGTATTAACAAGAATGAA

CACTGTAGCTAGTAACTAAAATTTTTTTCTTCATAAAAAATGGAAGTGTT

TTTCAGAATTCTAGGAAATGTCAAAGTAATTTTGGCTGTAGTCATATTAC

ATTTAATGGTATAGAGAATATTTCTAAAATTAACAACAATATTCTGTAAT

TTCCTCAGTTATTGCTACTGGTGATGAGGGATATAAAGTTGGCATGCTTT

CATGTTTTGAGCATCATCATTATACACTTCTGATTTTGCAAGTCAGAAAC

AATCTTCGTTTTAGTTAAGATAGCTGAGAACATTCTGAGGCAGGGGGTCA

GGCTTTCATTATTTGCATATTTAGGTAAGTAATATATAGACTACAATGAA

TTGATCAGAGTAACTACATATATAATTTTTAATGAAAGAATCATACAGTA

TTTATACATTTCTGTGACTTTTTAAAAGTTTATCAGAATATGGTAGTTTG

GGACCAAGGGCATAGGTTTTATTTCTGTATAGATCATTTTATTTTGTTAT

TTTCTGTGACCACTGGTAATAATCATCTTACAGATGTAAGCTGCCATTCA

CTGGGGAGCTTTGGCAATAGAATTGGCTAGATCAGCAAGCCCATGTCAAC

TATGGAACAACAACTTGCAGCTCATCTCTATTGATGGTGAGTCAGTGTCA

TCTTAATACATGAAGGATCATGTTAATGTTATTTTATCTTTGCAGCGTTA

TCAAACAACAGTTTGTCTTAAACTTTGTTTTATGAGACATTATTGGAATG

TTGAACCATGATGCTTCATTACTTGAACCCAAAGGACAACAAAGAAATCA

GCACAACAGTGAAAAATAAAGGAAGTTATAATTTGTTATCTGTGAATATA

CTTAAAATTTGTTTGAGTAGCTTCTGTCATCTCCATTCTCATTCCTTATA

ACACATGCCAGCTAACTTGTAATTCATGACTCTTATTTGAATACATACTG

ATTAGAAAAGAGTGATATTGATCACATATGTTGTATACTTATTTTAAAAA

TATATTACTAGGCCAGGTGTGGTAGCTTATGCCTGTAATCCCAGCACTTT

GGGAGGCCAAGGTGAGATCACTTGAGGCCAGAAGTTCGAAACCAGCCTGG

CCAACATGTTGAAATCCTGTCTGTACTAAAAACACTTCACTTGGGAAGCT

GAGGCACGAGAATTGCTTGAATCTGGGAGGCGGAGGTCGCGGT

>canFam2_dna range=chr3:18628073-18629932 5'pad=300 3'pad=300 strand=+ repeatMasking=none

TGTTTACAGGTGGCAGTGGGCAACATCATGGGTTGTAGCAGATGTTCTCA

ACATTTCTTTTGAAACTTCTTTATATTGCCTTGCAAGAGGAGCCCTAGAG

CCTCATCCCTGGGGTAGCTACTATGCTCTGGTATGATGCTGTGCTGTTCT

ATTTCATCCTTCCTGTTCTAGCAGAGGATCTAGGTTAGAATTTAGTGGTT

TCTTATTGCAGACAATTATGTAGAACTGGAAGTGGAATGTTTGAATTATA

CTGATATTGTGATTTCTTTGAGATTGTTTATTATGCCTTATTATTCTTAT

ACTTGTTCACAGAACCCCCAAATAGTGAGTGCCTAACTCATCATAGATAC

TTAGCATTGTGATTTTGTTTGAATTGTATCTAGTTGTAAACAGTTCCTTA

CTGATTTTTAATAGATGTTAATCTAATTTTTATTCTTTTAATTGATAAAG

AAGCACATAACATCATCTTTACTTCTACTTAGAAAAACTATGTCTTCAGG

TAATGTTTAACCAGTTTCTCAACTCTTATGTAGGAGATTCAGCTGGCAAC

CCATAAAATTCTATTTCAGGAATATTTAATTATAAACCTCTGTCTATATT

AGCATGAATAAACACTGTAGCTAGTAATGAAAGTATTTTTCTTCATACAA

AATAGTATTTTTCAGAATTCTGGGAAATGTCAAAGCAATTTTGGCTGTAG

TCATGCTGCATTTAATGTTATTGAGAATGTTGCTAACATTAACAAAAATA

TTCCGTAATTTCCTCAGTTATTGCTACTGGTGATGAGGGATGTAAAGTTG

GCATGCTTTCATGTTTTGAGCATTTTCATTATACACTTTTGATTTTACAT

GTCAGAAGCGATCTTTGTTTTAGGTAAGATAGCTGAGAACATTTTTACTT

AGGGATGGAGGTCAGGCTCTATTTGTATTTTTAGGCCAGTGCCTTATAGA

ATATAATGAATGTATCAGAGTAAAAACATGTACAGTTTTGTATGTAAGTA

TTATAGCATTTATGTATTTCTTTGACCTTATTTTTAGTTCATTAAAACAT

AGTGATTTGGGACCAAGGCATAGATTTCATTTCGGTGTTAATAATTTTAT

TTTATTTTCTGTGACCACTGGTAACACTCATCTTGCAGATGTAAGCTGTT

GTCCACCAGGGAGCTTTGGCAAGAGAATTGGCCAGATCACCAACTTCATC

TCCACTGTAGAAAAACAACTTGAAGCTCATCTCTGTTGAAGGTGAGTTAG

TATCATCTTCACACAGGAGAGATCATGCTAGTGTTATTTGATGTTTTCAG

GGTTATCAAGTGGAAGTTTGGTTTAAACTTTGTTTTATGGGATATTGAAC

CATCATATTTTATTACTTGAGTGTAAAGATTCACACAGAAATCATCAACC

TGACAATAAAAAGGGATGGAAGTAACAATTTGGTGTTTATGATACTCCTA

GAAGTAGTTTGAATTCCCTCCCTCCCTCCTTCCTTCCTTTTAAATGTTTA

TTTTTAAGTAATCACTGTACCCAGTATGGGGCTCAAACTCATGACCCTGA

GATCAAGAGTCACATGCTCTCCCAAGTGAGCCAGCCAGGTGCCCCAATTT

GGGTATTCTTTCTCACCTCCATTCTTGTTTCCAGATAATACATGCTAGCC

AAGTTATAATTCATGACTTTTATTTGAATGAATATTGGCTAGAGCAGAAT

CACATATGTTATATATTTATTTAAAAATATATTATGGATTTCAGGAAATT

TCATATAACTGCAATGCCAATATAAAGATACTGTTTTCAAAATCATTATT

TGTTCTTTGAAATAGTAGTCTTTATTAATGCTGGCCACAGGCTTGCTGTC

TTGGAGACAT

>rn4_dna range=chr2:5856978-5858847 5'pad=300 3'pad=300 strand=+ repeatMasking=none

TCCTAAGAGTCACAGGCTTGTGGTCAAAGGTTTGTATTTCTAATGCTTTC

CTATGTGATTACATTACTAAAGTACATAGAATCATATTGGCTTACTACAA

ATGCCAACTATTTTAAATCTTTCAGAACATATAAATTCAGTCCTCACGAC

AGTATATTCTACCTCATTAGTTACTAATGGCTGAAACTTAGAATTTGTTC

ATCAATGATATTGATGTTGCACAGCATTAATTCTACTCATAATACTTTTT

AGTAATGTCTACTCCTCTCTCCAACCATCATGAACTAGAAACCAGAAAAA

ATTCTATTTCAAGAATTGGTAGAAACACGCATCTCTACTAAGGAGAGTGA

ATAAAGTAGCTGAAAATTAAAGTACTTTTTCTGTATGAAAAGTGGGAATA

TTTTTCAGAATTCTGGGAAATGTCAAAGTAATTTTGGCTGTAGTCATATT

GCATTTAATGCTATAGAGAATATTGCTAACATTAACAAAAATATTCTGTA

ATTTCCCCAGTTATTGCTATTGGTGATGAGGGATGTGAAGTTGGCATGCA

TCCATGCTTTGAGCATCACCATTAGACAGATCTGATTCCACATGTCATAA

ACAATCTTTCCTTTTAAGACAGCTGGAACATTTTCAGTTGGAGGTAGAGG

TTAGGGTTGATTATTCTATATTTGCATCAGTGATATCTTAGAGTATAACA

AATGTACCAGAGTAAACACAAGTGTAAATTTTTATATATAAGACATTTTA

AAAACGTATCTGTGATTTTTTAAAAACTTATCAGAATATAGTAGTTTGGA

ACCAAAGGCAAACTTTATTCTATATTAATCATTTTATCTTTTATTCCTGT

GACATTGGGAACACTTGCTTTTCAGGTATTTAGCTGTTGTTGAGGAAGGA

GCTTTGGGAATAGAAGTAACCAGGCAGCAGGTCCATTTTCACTGTTGAAG

GCAGCTGAAAACTCATCCCTACTGATGGTAAGTGTCTTCTTCATGTGTGG

GAAATGGTATAAGACTACTCTGTTCAATGGGACCCTACTGGGATGTTGAA

GTATGATGCTTCATTGGTTAAGTGTAAGCAATCAAAATAAGAGTGAAAAA

GGAAGACCTGGGGCTAGGCAGATGCTCATTGGGTAAACTATTTGTGGAAT

AAACTTGAAGATCCCACAGGAAAGCCAGATATGGCCAAGTGCCTCTAACC

ACAATTCTGATATAGAAAGGGAGCTTACTGGTCAGGGACTAGCCAAAATG

ATGAGCTTCAGGTTTGCTGAGAGAACCACCTATCCCAAAAAATAAGGTGA

AAGGGCTGGAGAGGTGGCAAAGTAATTAAGAGCACTGGTTGATCTCTCAG

AGGAGAACATGGGTTCAACTCTCAACACCCAGATAGCAGCCAAAAACTAT

CTGTAACTCCAGTCTTGGGGGATATAATGCCTTCTTATGCCCTCAGTGGA

TACTGCACATATATTGAGCACATACATACATTCAGGCAAACAAACATTCA

GACACATAAAAATAAATGAAATCTCAAAGTATTTTAAAAAGATAATGTAG

AGGTCTGATATCAATCTTTAGACTTTGCAAAATTAGTTTTACACACACAC

ACACACATAAACACACATACACACACTATACATACACAAACACACACATT

TGAAAAATGTTATTGCTTAAAATATTTTAGATAACTTGTCTTAGTATTTT

TAATATTGATTTCATTAATATATTTATGTTGAGTCTAGGGTAAATCTTTA

TGCTGGAACTAGACCAGGATTATTATAAGATAAATTTAATGTATAAATTT

GAGTTCATAGTGGCTAGAATAATTCTTTTATGATTCTGGTAATAAAGTAT

CATTATCGTCTTAGATCTAA
