# Supplementary material for: The novel lncRNA lnc-NR2F1 is pro-neurogenic and mutated in human neurodevelopmental disorders
Source: eLife. 2019 Jan 10;8:e41770. doi: 10.7554/eLife.41770 (PMC6380841; doi:10.7554/eLife.41770)
Supplement: Supplementary file 7. [file elife-41770-supp7.zip › candidate_human_blincRNA_sequences/h.ncRNA_G_v1.rtf]

>NR_102706.1ggagccccgccccgaagcacagggtcgagtcccttctttccgctccaacgcacggagggtgaggtcggtacgcggtggtggcgtcacggcgccagctcctcccgacgccgagaccatgggggctcagctaagcggcggccgcggcgccccggagcctgcgcaaacccagccccagccccagccccagcctgcggcgccggagggcccggaacagccccggcatccgccccagccccagccccagccccagccccagccccagcccgagcccagcccgtgggggccgctggacgacgtgcgcttcctcatcgcctgcacttcctggtactgacggcgtcctccgcaggatgtcgcccgtctgtccgccgtcccctgtggttcttgcctgccttgtctcctctccccacgtccctgcgtctcttacaccccctcccacccgaggctccccagagatagcagagaattcgaagaggtcgccggggactggaaagaagtcccggcagggccgccttcgcagtctacaccccagcctgcttcccagcctacacccagacccagctcagaccttcgtgaccaccccatccctttctccggctggctgggtcgggggcatccctctctgtcgctggcttccagaggcaggacaggcctcctggtaagcccgcaaagttgctgacctcctgacttcgtctgccttttattaatatctgtattgctgataaccgtgctcttgactatgtgtcccaggtcatgtcccaggtcatggagaagcccgtgccacagtgaccctccccatactcctgggggggctgctctccatcctggatcgtaaggaggcatcatcaggctgtgttcctggaaccccaataaccctgggcccccagggccagcctgttgtagagggaggctatctgaccgccggtctggcagaggagatgggtgggcagctcccagacaccccaaaggacccggttctcttcccagagcgtcctaaggttactcttggaacctgatctttgttccctcatcccagggaaatgacacactctgtatttctgttttatttagaaatgatttaaaaaacattatacaaaggctgatcagtttaaaatgtgactgacactgaaatgctgtgatgtcccccaggctgaggggaagctaggctctggggcccccagtgctttgcccctctgtctgccctgtcctggggtgatggacaaacagatgaccacaggcaggagaatctgagattggaagcctctaggctgagccctctgggcctggccccacatccctcacctctgcagcctgggctgcctgcctccatctcctgttcattctcagctggcctgccaggagccaatggggagcctggcgggaggcgggggtgcctagagctttcaagaagtgagagcaccaacctgaggagtggacagggaccaggaagtgggggaagggaggccaggaagaggtggatacaggagacacttctcatctcatctcagaccctagaggggtccacagatggggacacaagacccagccagcccactggatggcccgggcaagtaacaacctctctgtgcttcatctgagggcacggtgagagttaccgtcggcctcccagggcctaacacgagtttcatgtgagtggacaggtgtgagctaataaagtgctttgcaaagtataaaacactgtacaaacctatgaaaaaaaaaaaaaaaaaa
