# Supplementary material for: The novel lncRNA lnc-NR2F1 is pro-neurogenic and mutated in human neurodevelopmental disorders
Source: eLife. 2019 Jan 10;8:e41770. doi: 10.7554/eLife.41770 (PMC6380841; doi:10.7554/eLife.41770)
Supplement: Supplementary file 7. [file elife-41770-supp7.zip › candidate_human_blincRNA_sequences/NR2F1- AS_I_NR_109825.1_sequence.rtf]

>NR_109825.1tgagctccaaggtcgtggtctggtcctcagggatgaggtcccgggtccagactctttccttggtcagcaactcctgcttagaagctccccgccccgaatggaatcgtgacctggcgctaggtcccccgctgggagtgcccgaggctttcctgaaggccccaggccttcaggccgccagggtcctaaggtgcctgtggcggcccctcgtccgcctgagccctgagatcagcgcggccctcggtgctgcagggccccagggcggccaggaaacacctgcccgaagtccaggcccggccccactgcagtccaggaccctaattctcgagtgtctcttcacgaccctgaccccggaagggcgctcccttgcgaacgaatttttaattctgcggactgggatcccttgtaggggcccctccgagggcccctcccacccctttccctggactagcgaactccgaccctaagcgcggccccgcagggagcggatcccggaagctaggcccatatccggccgcggagccttgcgcgcggggttggctgccgggaggtcgacgctagtgcggtcccccgcgcggcggaggggcccgggccgcggtatctgcgcactgagacgtcggcggaaggctagaggccgtgctctcgcccccgcagaaggccaccactccttgagaaggccggagggacaaagcaccgggcctgcgccgggggcattcaagaaggaagccaggcccgaccgggggtggcgcacgcttcccgaaccccaggcccacggcccggttccgcgcgggcagttggcgccccctcgggtcggcacagcagacctcttagtaatggtccccagggagtacagggactgagaaacggaaatactaccatcctccagccaatctgttgggaggactgcttgcagaggactcgtgctccagatgttgcactagaggcgctgcaagtgtgagcagcaggtagatgaaactcaagagaaaaggtgtggataaatgaaactagcccatgatgaacctgttttctccgtgaccacaatattaaccaggatgaatggcggtggcagtgggcctgcatcacaggttgcagcagatgttctcaatatttctattaaaatttccttatttccatatgcaagaggagccccagagctgcatccttatggtagctaccatgccgtgatgtaagctgccattcactggggagctttggcaatagaattggctagatcaggaagcctatgtcaactatggaacaacaacttgcagctcatctctattgatggacctctaacaatgatattggaagaatagatactaagtcttggagaggtgaaacagtcgcaaatggaattccaagaaacattctatcatcaggtattatctactgctgtctgcagaattcagacatggagggctcaatggtgtcccctggtctttggaacattctgtattttaatgatcatgatacactgcctctaggactgggacagatagccactgagtacctgcttcaaatcaccctgcacgttttctctttggaaagataagcactggagaaccatctcgttaccttcagcagaaatggaaacaaatcaagacattaagaatgccgcagtggacagcagttatgcaaggttcagcagaggaagttttcaagatggtgtgcctcactagccagaaatcagaaaagatcgaagctagccttttcatctcagctgctccttaaatcagtgcaacccctccaaattaatgtgaattgaaacttgcattgcttttcaacttaattgaatacttactatatgtgaaccactgcacaaaataggggaagaagatgtgggagacaaagatgtggagcctgtcttcaataaatttacagttagtttcagggtacattgtttttcttctgctgttaattaggtgtatccccaagacaataaatacatgaatggaaaagtatattctgtgttttaagtctctttaataaaaaacaaaacaaaatccaaaaaaactcctaacatggcaactcaggacattttaatccttttttttgttgttgggcttctgataggatccattttattgattccataaatatttagtagactttagacatattaatacattgtagtttgagttaatttgcattaaattctgtttttttccccttaggtatagattgtctgttggaaaaatatattgcttgtggatttcttggggagtgggaaggtgaattatacctgttgtctctacagtccactaccttccacaccctagggcagtggtccccaaccttcttggcaccagggatcagtttcctggaagacaatttttctgtggatggtgtggggatggttttgggatggaactgttccacctcagatcatcaggcattagttaggctctcataacgagtgcgcagcctagatccctcacatgcacagttcacagtagggtttgcgctgctatgagaatctaaggctgccactgatctgacaggaggcagaactcaggtggcaatgcttactggcccactgctcacctcttgctgtgcggcctggttcctaataggccatggacccccacagacgggtactagtctgcagcctgtggaccccagccctaggggcctgccattctgagtgagttattagcatgaagaagaggaagatctggatcaaacgtgggtgatggtactcagagacccaagataggatagggatggaacaaaatccatctgggcaacggctctatgaaaatttggctaagtaattatttcagtattcattgtattcattgtccttcgtgttccataagttaaatgactgtctaatttttccaaaaatttatttctgacttgagaataagtgtgtcatgattttcccagtgtaaagacactgatataactgtagataccagacattttatgtagtgtctatgacacattttagtatgtatgagccaacaatagacatgtctttgtcttgaggagtgtccatctgaattgaaaatgtgtcagcttttttttaacatcatcaacagacttcttaattaagctgccaatacatactgccaatagcactgtgtgctgtctgagaaatgcattgtgtaagtgctatttccatcttattaaataaacaatgttgctctgtataaactggatttaaagtgttctttgaatgaggcattatattgctccataaataatgtgatgtgaattctcatatgatcccttttaacttaatttctaaaaaaatattatttgctcctcagtaaggaatagctctaattggactggtaactttagtgatatgatttgtctgtaacaggctgaatcgccccactcccaaactcctggtcgtgatactggatatttgttatgccacttttagtttgcatgaggtatcttccaggaagtattagttattaacttgtgttctactaaactgtagatatttatttcctgttctggaagatttcttggagtgtccatctgtgcattcctggtgcctagtgtagtcttgctaatggaatagcacctttattggtgctacttaggggttggtagcttacaatcaatctacttggaacgtatccaaataaactggatatttttataca
