# Supplementary material for: The novel lncRNA lnc-NR2F1 is pro-neurogenic and mutated in human neurodevelopmental disorders
Source: eLife. 2019 Jan 10;8:e41770. doi: 10.7554/eLife.41770 (PMC6380841; doi:10.7554/eLife.41770)
Supplement: Supplementary file 8. [file elife-41770-supp8.zip › candidate_mouse_bliNcRNA_sequences/ncRNA_V_sequence.rtf]

CAGCAGCAGCAGCAGCAGCAGCTTCCAGACATGAGAAGTGTGGTTGATCTGAGTTTGGGTTTTATTTGGGTTTTTACTGTTCAGGTGTAACTGATGGCATATCTGAGGGGGAGGTGGGGAGAGAGAGAGAGAGAGAGAGAGAGAGAGAGAGAGAGAGAGAGAGAAGAAAGACTGACTGACTGACTGTAAAAGCGACTCACAAGCGCACCCTGCCTTCTCTGGCCACGGTCTTACTGCAGTAGTCAAGCATTTGTGCCGTGATTTTCTGGATTCAGAAAGAGCTGATGTCCTCAAGAGAGTTGAAAATCCAGAGCCCTGCAGCTTTTCCTAATTATCTGTCTTTTCTCTCTTCTCCATCACCCAGGGGACAGAAACACTGCTCATCACACAGCCTGCTTGAAAGAGTTTCCTGTCTTGTGCACATTATCCCTTGATATTGAAATTATTTTCTCTGTTTAATCCGGCTCCTGCTGAGAAGCTGTGTGGGATTTAGGGTGCAGGGTTTGCTCTTGTACTTTGCAGTGGGGTTGCATCTTTTCCTCCTAACCTGAGGGTAGGTCTCAGCTGCTGCCGTTCCGTTTCATGCAGAAGACAGCCTCTGATCCAGGGAATTTTTTCAGAGTTCTGACCTGATCAAGGGTCCCTACACCAGTCTCCTCAGAGCCTGGGTGAGAAGAGTGGGCTTGGATAAGAGGTCTCTGGTTAGGAGAGGGTCATGTTCCCTGGACATCCATGTTACCTTGTTTCCAGGGTTTTCCACATTGAGGAAGGGGCACTTGCCATGCTGAGCGTCAAATCTGAAGACAACTGTACATCTCCCCAGGCTGCCCACACTTCTTCACGCTGCTGTTTGGACCGCGCTTTATGATTCAGAGGACCAACCACCTAAGCATAGAAGGACTTGGCTTTCCGTTTGAACTTTGACAGAGGTTCTGTGATGTTTGAATTTTCTGTCTGTCTCTTGCGCAAACCCAACAGTGTATGTAGTCCCTTTGGCCCCCTCCACCCCAGAATATCCTAATAAACAC
